# Neonatal Necrotizing Fasciitis of the Foot Caused by Bacillus cereus: A Case Report

**DOI:** 10.7759/cureus.74031

**Published:** 2024-11-19

**Authors:** Ayana Kitta, Takashi Saisu, Jun Kakizaki, Yasuhiro Oikawa, Yukie Metoki, Ken Okazaki

**Affiliations:** 1 Department of Orthopaedics, Tokyo Women’s Medical University Yachiyo Medical Center, Yachiyo, JPN; 2 Department of Orthopaedics, Chiba Child and Adult Orthopaedic Clinic, Chiba, JPN; 3 Division of Orthopaedic Surgery, Chiba Children’s Hospital, Chiba, JPN; 4 Department of Orthopaedic Surgery, Kitasato University School of Medicine, Sagamihara, JPN; 5 Division of Orthopaedic Surgery, Tokyo Women’s Medical University, Tokyo, JPN

**Keywords:** bacillus cereus, fulminant neonatal necrotizing fasciitis, neonatal musculoskeletal infection, osteomyelitis of the calcaneus, septic ankle arthritis

## Abstract

Few neonatal cases of soft tissue and osteoarticular infections with *Bacillus cereus* have been reported. We report a rare clinical presentation of necrotizing fasciitis of the foot caused by *B. cereus *in a 17-day-old male neonate with hypoplastic left-sided heart syndrome. Fulminant progressive black skin necrosis was triggered by fresh frozen plasma leakage from the peripheral venous access. This patient was treated with antibacterial agents as soon as skin lesions appeared, and the necrotic tissue was debrided in 48 hours. The patient recovered after three months of hospitalization without any subsequent complications in the 3.5 years of follow-up. The immune system is not fully developed at birth, resulting in high susceptibility of neonates to *B. cereus* systemic infection. When a neonate presents with a rapidly progressive necrotizing soft tissue infection, *B. cereus* should be considered as the possible cause and treated promptly.

## Introduction

*Bacillus cereus* (*B. cereus*) is a gram-positive, spore-forming rod, which is widely distributed in the environment [1]. Although *B. cereus* is a rare human pathogen, except in cases of food poisoning, neonates and premature infants with weakened immune systems can develop systemic infections resulting in death [2,3]. *B. cereus* is associated with a range of infections, including sepsis, infections of the central nervous system, intraocular inflammation, pneumonia, and skin infections resembling gas gangrene [1]. Few neonatal cases of soft tissue and osteoarticular infections with *B. cereus* have been reported [2]. In this study, a case of neonatal necrotizing fasciitis of the foot is reported.

## Case presentation

**Figure 1:**
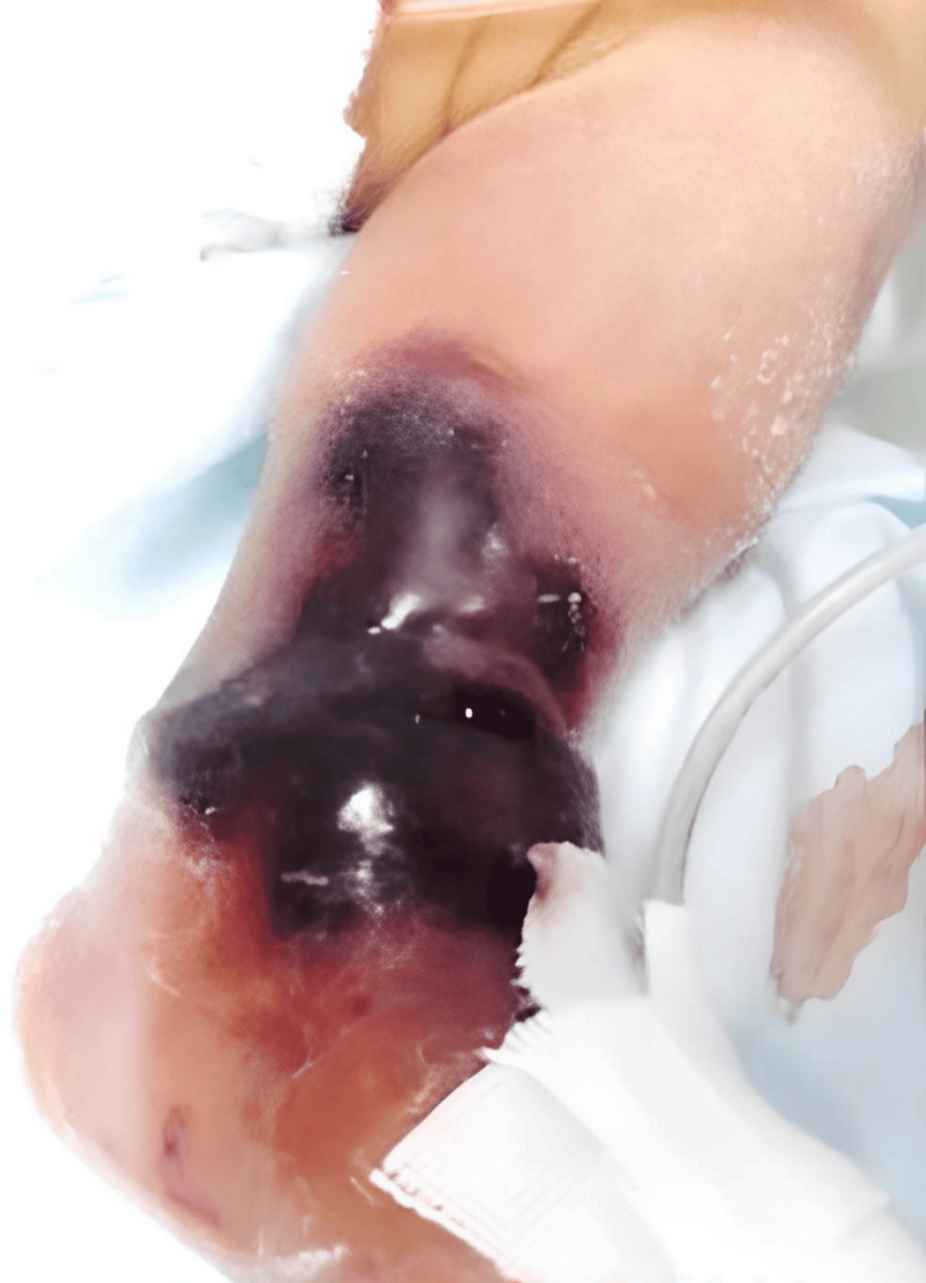
Blackened skin on the patient’s left foot.

The patient was male, born at 38 weeks and 0 days through vaginal delivery, and weighed 2,479 g. At the age of nine days, the Norwood procedure was employed for hypoplastic left heart syndrome. After surgery, the patient was intubated and admitted to the neonatal intensive care unit (NICU) for general care. On day 17, fresh frozen plasma leaked from the peripheral venous access to the back of the left leg, which resulted in fulminant progressive black skin necrosis and blisters (Figure 1).

**Figure 2:**
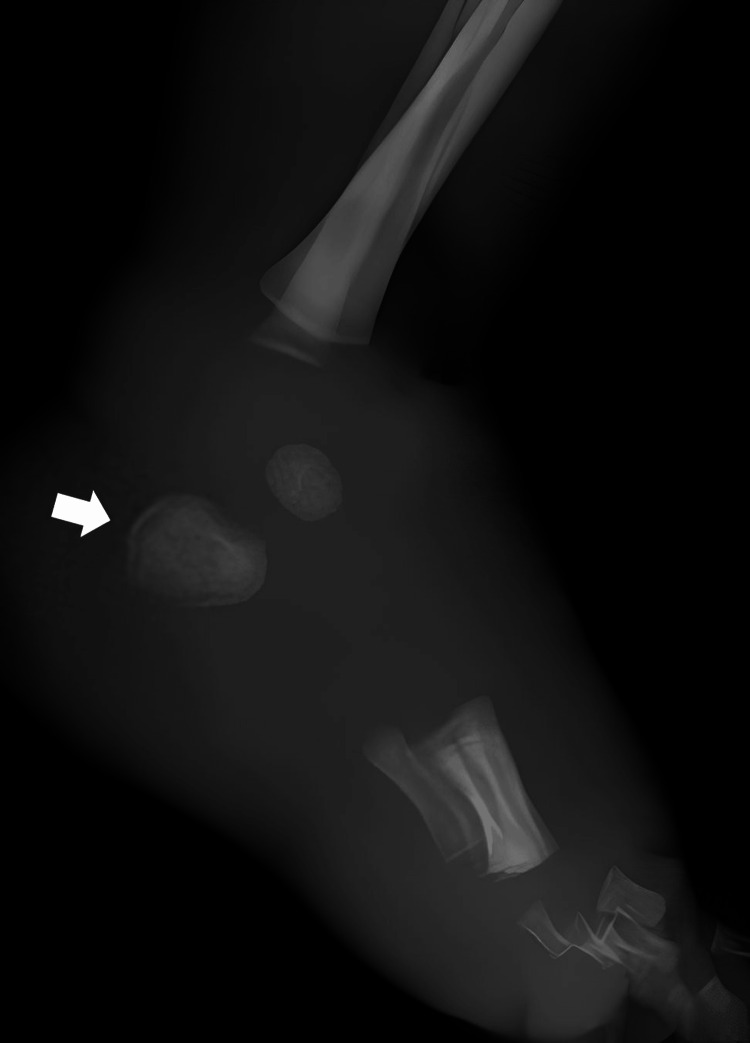
Radiograph showing osteolysis at the base of the calcaneus. The arrow points to the calcaneus.

The necrotic tissue was debrided by a plastic surgeon; no fever or significant changes in vital signs were noted. A white blood cell count of 10,100/μL, with 93% neutrophils, and a C-reactive protein level of 10.45 mg/dL in the blood were indicative of a strong inflammatory response. Abscess and ulceration were observed in the central venous tract extraction scar on the right side of the neck and in the inguinal region. The blood and skin from the central venous tract extraction site and the left foot were cultured. The following day, the patient was referred to our department because of rapid expansion of redness, swelling, and heat over the entire left lower extremity and groin. The patient had a fever of 37.5°C and a stronger inflammatory response, with a C-reactive protein level of 19.20 mg/dL. *Bacillus* spp. was isolated from the blood cultures. As osteolysis of the left calcaneus was identified in the radiographs (Figure 2), we diagnosed osteomyelitis of the calcaneus.

**Figure 3:**
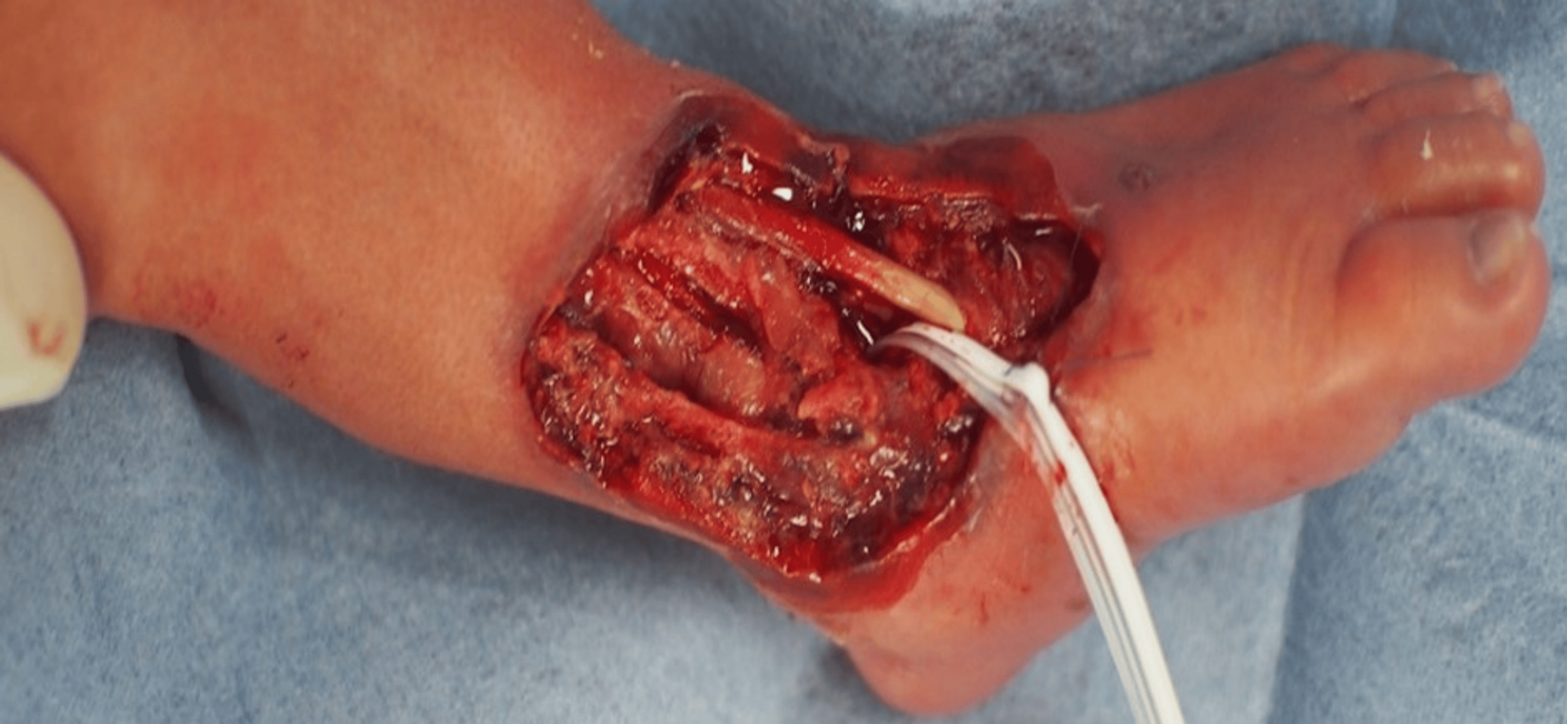
Postoperative photograph.

We decided to perform surgery to remove the necrotic areas of the skin and subcutaneous tissue anterior to the ankle joint 48 hours after the skin lesion appeared. A large amount of purulent joint fluid was drained through an incision in the ankle joint capsule. Cortical fenestration of the calcaneus was performed, and no obvious purulent discharge was observed. After lavage with a large volume of saline, a drainage tube was placed within the ankle joint and under the calcaneal periosteum (Figure 3).

*B. cereus* was isolated from the blood and left foot skin cultures submitted on the first day of illness. Thus, the patient was diagnosed with necrotizing fasciitis caused by *B. cereus* with pyogenic ankle arthritis and calcaneal osteomyelitis. Range of motion (ROM) rehabilitation was initiated on the third postoperative day, and splint immobilization was performed on the fourth postoperative day to prevent equinus foot deformity. Two months after surgery, the patient had regained full ROM, and the splint was removed.

**Figure 4:**
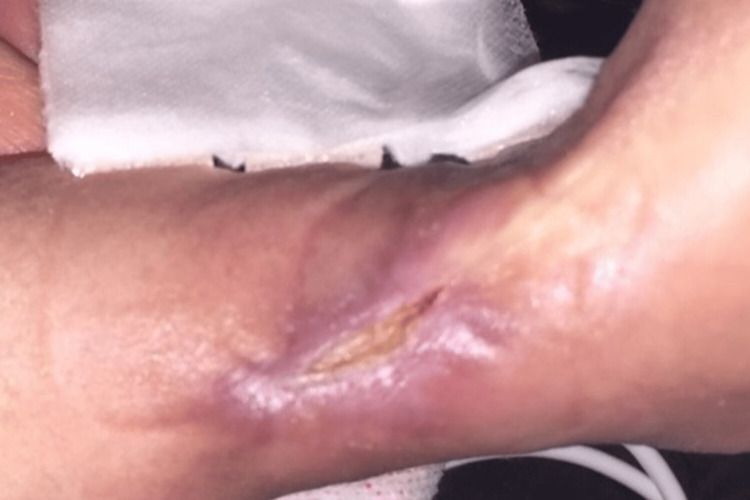
Photographs after negative-pressure wound therapy. Two months after surgery.

Ceftazidime (200 mg/day) and vancomycin (60 mg/day) were administered from the first day of tissue necrosis. On the second day, *Bacillus* spp. was isolated from the blood and skin cultures, and thus ceftazidime was discontinued; the patient instead received vancomycin and meropenem (100 mg/day). On the fourth day, the blood cultures were negative; hence, only vancomycin was administered for six weeks. The drainage tube was removed six days postoperatively. Negative-pressure wound therapy was performed from the 10th postoperative day, and epithelialization was observed within approximately two months (Figure 4).

**Figure 5:**
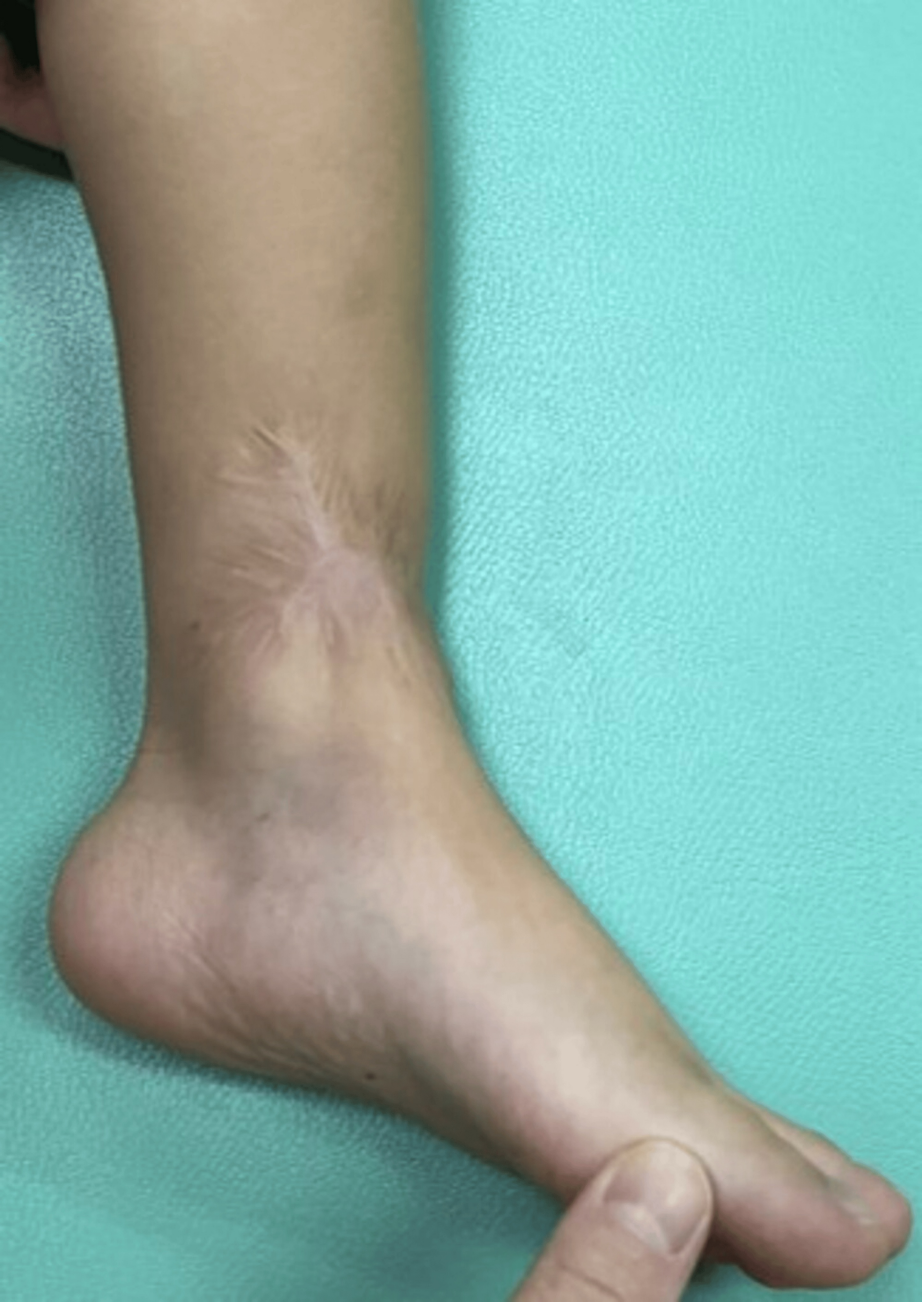
Photograph at the last follow-up.

The skin lesions and inflammatory response improved gradually, and the patient was discharged at three months and 10 days of age. At the latest examination, 3.5 years after surgery, no skin contracture in the scar area, limitations in ROM, pain, or difficulty in daily living were noted (Figure 5). Radiographs showed no obvious abnormalities, except for early ossification of the distal tibial epiphyseal nucleus.

## Discussion

*B. cereus* is a gram-positive, ubiquitous bacterium that is present in soil and sewage and is resistant to heat, alcohol, and detergents [3]. Although it rarely becomes a human pathogen, it can be transmitted to immunocompromised patients as an opportunistic pathogen. *B. cereus* is a risk factor, especially for infants, intravenous drug abusers, and patients with trauma or who have undergone surgery and have indwelling catheters [1]. *B. cereus* causes a variety of infections, including sepsis, central nervous system infections (e.g., meningitis and brain abscesses), intraocular inflammation, pneumonia, and gas gangrene-like skin infections [1]. A particularly high mortality rate has been reported in premature infants [2]. Moreover, *B. cereus*-infected newborns have been reported to present with bloodstream infections, necrotizing pneumonia, enteritis, and meningitis with brain abscess [4,5]. In addition, a pediatric case of osteomyelitis caused by *B. cereus* has been reported [6]. Here, we report a case of necrotizing fasciitis with septic ankle arthritis caused by *B. cereus*.

Saikia et al. reported clustered anthrax-like skin infections caused by *B. cereus* in 12 healthy newborns. They presented with extensive bullous skin lesions, two of whom developed necrotic lesions within hours of birth. All patients were treated with antimicrobial therapy [4]. Viel-Thériault et al. reported a case of fulminant necrotizing soft tissue infection caused by *B. cereus* in an extremely low-birthweight infant who died [2]. The patient was born at 22 weeks of gestation, weighing 500 g, via vaginal delivery. At five days of age, a necrotic skin lesion appeared on the left shoulder which was consequently treated with antimicrobial agents. However, the skin lesion worsened rapidly, and the patient died the next day due to the septic shock. Because of the risk of rapid deterioration in an immunocompromised host such as a low-birthweight infant, aggressive surgical debridement should be considered in addition to antimicrobial therapy. Patients with reported *B. cereus* infection were treated with antimicrobial therapy, which included vancomycin, cefotaxime, and fluconazole after the appearance of skin lesions [2], or with ciprofloxacin and amikacin [4]. *B. cereus* produces β-lactamase and is resistant to penicillin and cephalosporins. Susceptibility to aminoglycosides, clindamycin, vancomycin, chloramphenicol, erythromycin, and fluoroquinolones has been reported [7,8]. If *B. cereus* infection is suspected, immediate antimicrobial therapy with a broad-spectrum antibiotic is recommended. In addition, the results of an environmental study by Saikia et al., who reported an outbreak of anthrax-like skin infections caused by *B. cereus* in newborns, showed that medical instruments used during delivery were contaminated with *B. cereus* spores. The skin lesions resulted from micro-skin abrasions that occurred during delivery or when washing the baby [4]. In a report of a *B. cereus* outbreak in a NICU, the bacteria were detected in a balloon used as a respirator [5]. Furthermore, *B. cereus*-contaminated hospital linens have been reported [9]. In the current report, there was no similar *B. cereus* outbreak and no environmental survey was conducted; however, measures such as environmental surveys and infection control steps should be conducted in the event of an outbreak of *B. cereus* infection.

## Conclusions

In our study, the patient’s skin was debrided within 48 hours after the appearance of the skin lesions and progressed well. Negative-pressure wound therapy effectively managed skin defects following debridement. *B. cereus* is widely distributed in the environment but is rarely considered clinically important because of its low virulence. Nonetheless, without adequate treatment, *B. cereus* infection in neonates can be fatal. In neonates with rapidly progressive necrotizing soft tissue infections, *B. cereus* should be considered, and prompt surgical treatment and systemic administration of antimicrobial agents should be performed.
